# Loss-Framed Adaptive Microcontingency Management for Preventing Prolonged Sedentariness: Development and Feasibility Study

**DOI:** 10.2196/41660

**Published:** 2023-01-27

**Authors:** Woohyeok Choi, Uichin Lee

**Affiliations:** 1 Information & Electronics Research Institute Korea Advanced Institute of Science & Technology Daejeon Republic of Korea; 2 School of Computing Korea Advanced Institute of Science & Technology Daejeon Republic of Korea

**Keywords:** contingency management, incentive, sedentary behavior, sedentariness, behavior change, health promotion, financial incentives, health intervention, user compliance, incentive-based intervention, mobile phone

## Abstract

**Background:**

A growing body of evidence shows that financial incentives can effectively reinforce individuals’ positive behavior change and improve compliance with health intervention programs. A critical factor in the design of incentive-based interventions is to set a proper incentive magnitude. However, it is highly challenging to determine such magnitudes as the effects of incentive magnitude depend on personal attitudes and contexts.

**Objective:**

This study aimed to illustrate loss-framed adaptive microcontingency management (L-AMCM) and the lessons learned from a feasibility study. L-AMCM discourages an individual’s adverse health behaviors by deducting particular expenses from a regularly assigned budget, where expenses are adaptively estimated based on the individual’s previous responses to varying expenses and contexts.

**Methods:**

We developed a mobile health intervention app for preventing prolonged sedentary lifestyles. This app delivered a behavioral mission (ie, suggesting taking an active break for a while) with an incentive bid when 50 minutes of uninterrupted sedentary behavior happened. Participants were assigned to either the fixed (ie, deducting the monotonous expense for each mission failure) or adaptive (ie, deducting varying expenses estimated by the L-AMCM for each mission failure) incentive group. The intervention lasted 3 weeks.

**Results:**

We recruited 41 participants (n=15, 37% women; fixed incentive group: n=20, 49% of participants; adaptive incentive group: n=21, 51% of participants) whose mean age was 24.0 (SD 3.8; range 19-34) years. Mission success rates did not show statistically significant differences by group (*P*=.54; fixed incentive group mean 0.66, SD 0.24; adaptive incentive group mean 0.61, SD 0.22). The follow-up analysis of the adaptive incentive group revealed that the influence of incentive magnitudes on mission success was not statistically significant (*P*=.18; odds ratio 0.98, 95% CI 0.95-1.01). On the basis of the qualitative interviews, such results were possibly because the participants had sufficient intrinsic motivation and less sensitivity to incentive magnitudes.

**Conclusions:**

Although our L-AMCM did not significantly affect users’ mission success rate, this study configures a pioneering work toward adaptively estimating incentives by considering user behaviors and contexts through leveraging mobile sensing and machine learning. We hope that this study inspires researchers to develop incentive-based interventions.

## Introduction

### Background

Intrinsic motivation refers to an inherent motive to perform a target behavior [1], whereas extrinsic motivation is a specific type of motivation for obtaining a certain outcome that can be separated from the behavior [2]. Although it is clear that intrinsic motivation is essential for behavior change, numerous studies have presented evidence that extrinsic motivation can also greatly contribute to behavior change. A representative behavioral therapy that uses extrinsic motivation is contingency management, which provides external rewards (typically financial incentives) contingent on the occurrence of behaviors of interest for reinforcing positive behavior change [3]. Such a therapeutic approach has already shown effectiveness on behavior change in various fields, including physical activity promotion and dietary tracking [4-9], prevention of drug abuse [10-17], smoking cessation [18-22], productivity and academic performance [23-25], and driving behavior [26,27]. In addition to its application in academic fields, companies also use financial incentives as core motivators for behavior changes, such as health insurance discount programs for healthy behaviors [28] and a commitment contract that allows the company to send money from a user’s account to a particular person or organization (eg, charities) if one fails to reach a self-defined goal [29].

However, the design of incentives in these contingency management interventions has several issues that hinder the achievement of the goal of these interventions, which is to promote successful behavior change. For example, socioeconomic status probably contributes to incentive effectiveness [30,31]. In addition, the assumption of a trade-off between ability and motivation (namely, users with low and high ability require high and low motivation for behavior change, respectively) may imply that the magnitude of an (extrinsic) motivator should differ by context and one’s physical and cognitive capabilities for eliciting behavior change [32]. Another aspect of incentive design that should be considered is the delay between behavior occurrence and incentive delivery, with a shorter delay having shown greater effectiveness in eliciting behavior change [18,33]. Other potential contributors to the effectiveness of contingency management include incentive framing (eg, providing incentives for positive behaviors vs deducting expenses for negative behaviors) [23,26,34,35], incentive magnitude adjustments throughout the intervention [20,34,36,37], and incentive magnitude certainty (eg, fixed vs lottery incentives) [4,12,38].

Although previous studies have shown the effectiveness of the incentive designs of their proposed contingency management interventions, they had several limitations. For example, positive behavior was not immediately rewarded, and only behavioral outcomes from long-term behavior adherence were rewarded at the end of an intervention (eg, lump-sum provision) [7,21]. In addition, incentive magnitudes were often fixed [23,39] or randomly sampled from a predefined range of incentives [4,38] (ie, they did not change by context at the individual level). Moreover, although several studies have proposed an escalating reinforcer where incentive magnitudes increase at each positive behavior occurrence [20,34,36,37], such a design requires intervention practitioners to configure a detailed plan manually (eg, the amount of incentive increment) based on their domain knowledge.

### Objectives

This study proposes a novel incentive-based mobile intervention named loss-framed adaptive microcontingency management (L-AMCM), which immediately discourages users’ microbehaviors that cause adverse health effects by providing a personally and contextually tailored incentive. In more detail, this approach delivers a prompt recommending a positive behavior change when the user is susceptible to health risks. Each prompt presents a particular expense framed as a loss (ie, a loss-framed incentive), in which, if the user does not change their behavior in response to the prompt, that expense is deducted from an individual budget that the intervention regularly allocates. In addition, the deducted amount presented in each prompt is dynamically adjusted based on individuals’ responses to prompts over varying incentives and contexts. To this end, the L-AMCM continuously monitors users’ behavior changes in response to prompts presenting varied contexts and expenses. It iteratively learns an individual’s behavioral model, which describes the likelihood of a behavior change in a given context and at a given expense. On the basis of the learned model, the L-AMCM estimates how much each prompt needs to bid to elicit positive behavior, at least to some extent.

To evaluate the feasibility of the L-AMCM, we applied it to a mobile health intervention app that delivers active break missions (ie, standing up and moving around for a while) with an estimated incentive via individuals’ smartphones to discourage prolonged sedentary behavior (ie, 50-minute uninterrupted sitting sessions). This study illustrates the lessons learned regarding its application via a 3-week field study with 41 participants. We hope that this study will provide new research directions for incentive-based mobile interventions.

## Methods

### Design of the L-AMCM Intervention

#### Motivating Scenario

Herein, we illustrate an exemplar intervention scenario with microincentives in the domain of prolonged sedentary behavior interventions, similar to those in previous research [40,41]. When users uninterruptedly sit down for a long time, a given health intervention app triggers a prompt containing a behavioral suggestion for breaking the sedentary period (eg, standing up and moving around for a while) and bids a certain amount of monetary incentive that will be withdrawn from an individually assigned budget if users do not adhere to the suggestion. Users then examine whether the compensation is sufficient to make them adhere to the behavior suggestions in a given context.

For example, if they receive the prompt late at night, a period in which they may be feeling somewhat tired, they may choose not to adhere to the suggestion if the incentive is low; they would rather continue engaging in sedentary behavior. However, if the intervention bids a larger incentive, they may consider accepting the behavioral suggestion. In addition, if the prompt is coincidentally delivered immediately or closely after they have spent some time working very hard at the office and may be feeling the need to refresh, they may be more willing to take an active break even with a lower incentive. However, it is clear that the tendency to accept behavioral suggestions with incentives will differ by user. For example, users who already know the health risks of prolonged sitting sessions may be willing to try to comply with more active break suggestions even with lower incentives.

A core assumption of the presented scenario is that *users are more likely to accept the behavioral suggestion as the incentive grows*, which stems from the evidence of various studies showing that a larger incentive magnitude corresponds to a larger effect on health behavior change [7,10,18,31,33]. Another assumption is that *the incentive magnitude necessary for eliciting behavior change may differ by user and context*, which is grounded in the Fogg Behavior Model, a practical framework illustrating the underlying factors relevant to behavior change [32]. In this model, a particular behavior happens through the interplay of an individual’s inherent *motivation* toward the behavior; an individual’s *ability* to perform the behavior; and an external *prompt* that elicits behavior change by reminding the behavior, reinforcing motivation, or simplifying the behavior. In the presented scenario, the amount of incentive plays a role in sparking positive behavior change, and the change in incentive magnitude across users and contexts is based on several important aspects of the Fogg Behavior Model, as shown in Textbox 1.

**Key aspects of the Fogg Behavior Model considered in the proposed incentive mechanism.**

- Fogg Behavior Model key aspects
  *Motivation and ability have a trade-off relationship* (eg, lower ability requires more motivation for behavior change); thus, the amount of incentive necessary for the positive behavior might need to change across different levels of motivation and ability. For example, for people with enough adherence motivation, the amount of incentive required for the behavior change might be smaller compared with less motivated people. In addition, people with less ability might need to be compensated with more incentives for behavior change.
  *Motivation and ability differ among individuals*; thus, the amount of incentive required for the behavior change would be different by individual.
  *Ability differs by context*; thus, the amount of incentive required for positive behavior change might vary by context.

#### Hypothetical User Behavior on Incentives and Contexts

On the basis of these assumptions, we hypothesized an equation for a user’s behavior occurrence likelihood, *y* ∈ [0,1], with a given incentive magnitude *r* ∈ *R* and context *c* ∈ *C* as follows: *f* : *r*, *c* → *y* such that ∀*r*, *r’* ∈ *R* and *c*, *c’* ∈ *C* *f* (*r*, *c*) ≤ *f* (*r’*, *c’*) if *r*≤*r’* and *c*=*c’*.

Various functions satisfying the aforementioned equation can be used to model the hypothetical user behavior we propose. This study considers a logistic regression (LR) model as it naturally maps an input into a probability output and is easily implemented and interpreted [42]. Then, assuming a vector of one-hot encoded discrete contexts, *C*={*c*_1_, *c*_2_,..., *c_n_*_–1_, *c_n_*}, and the corresponding coefficients (ie, the effect of context on behavior occurrence likelihood), *B*={β_1_, β_2_,..., β*_n_*_–1_, β*_n_*}, the hypothesized user behavior can be modeled as follows: 
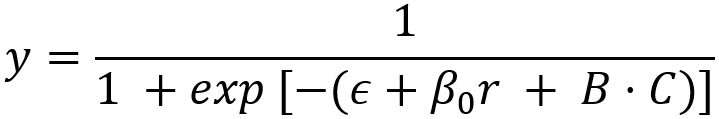
, where β_0_ indicates the effect of incentive magnitude on behavior occurrence likelihood and ∈ is an intercept term.

#### Estimation of Incentive Magnitude

The hypothesized user behavior can also be rearranged for estimating the incentive magnitude necessary to elicit a target behavior with a given probability, y ¯, as follows:
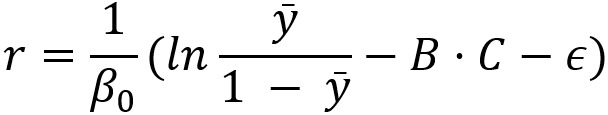
.

Importantly, incentive providers can choose the y ¯ depending on their policies. For example, if greater costs are not of concern, a large y ¯ will make users highly likely to comply with the behavior suggestions, whereas y ¯ close to a half probability will make adherence to behavior suggestions highly uncertain. In addition, β_0_ is assumed to be >0. A user’s behavior model with a nonpositive β_0_ implies that the incentive does not influence or even deteriorates behavior occurrence likelihood, and such a situation contradicts the assumption that larger incentives are more likely to elicit behaviors.

**Figure 1:**
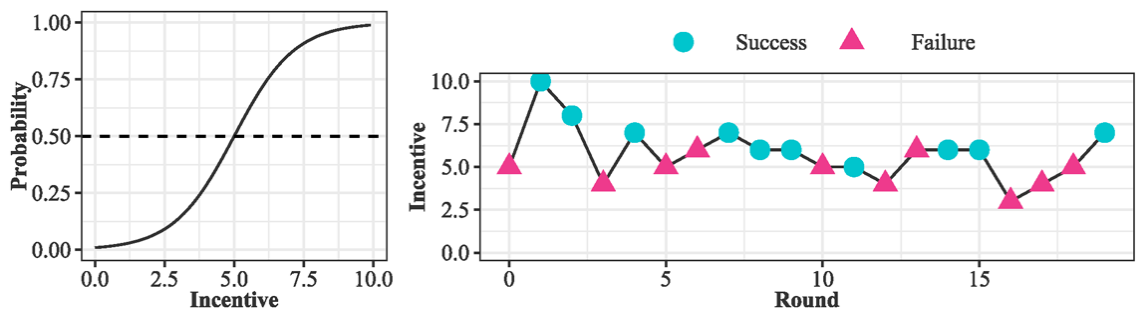
A toy example that illustrates how the proposed strategy works. The left panel shows a user’s behavior probability across different incentives, and the right panel describes a trace for finding a particular incentive magnitude that is necessary for eliciting positive behavior change. y ¯ is set at 0.5.

Throughout the intervention, the hypothetical user behavior is rebuilt after each incentive bidding and behavior occurrence observation; namely, the rejection or acceptance of a particular incentive magnitude results in gradual changes in the estimated incentive for the next bid (Figure 1). Accordingly, incentive magnitudes are dynamically adjusted by the proposed incentive estimation to find the appropriate incentive magnitude that can elicit behavior occurrence with a probability, y ¯.

In addition, we only consider recent behaviors for building the hypothetical user behavior (and estimating an appropriate incentive) as user behavior occurrence likelihood related to incentives may change over time. For example, initially, users may choose to adhere to a behavior suggestion because they know that they will receive monetary compensation for the suggestion, not because the behavior may improve health outcomes; however, as they adhere to the behavior because of the knowledge of subsequent compensation, they may eventually perceive the usefulness of performing the target behavior, thereafter potentially becoming intrinsically motivated to conduct the suggested behavior with little or no incentive [1]. Considering this, it may be that more recent response behaviors are more important than older ones in modeling behavior occurrence. A detailed algorithm for the proposed incentive strategy is presented in Multimedia Appendix 1.

#### Loss-Framed Incentive

A major characteristic of the proposed incentive mechanism is to bid higher incentives as the target behavior becomes less likely. For example, once a user rejects a behavioral suggestion for a given incentive magnitude and context, our mechanism would assume that such a magnitude is insufficient to elicit behavior change. Therefore, the subsequent behavioral suggestion triggered in an identical context will bid a greater magnitude. Otherwise, the user is offered the same or a smaller incentive at the next behavioral suggestion. Such a characteristic may yield gaming behavior if the user is rewarded for succeeding in behavioral missions (ie, a gain-framed incentive). For example, the user may deliberately reject the current bid suggested by the intervention prompt and maintain an unhealthy state to earn higher incentives at successive bids.

To discourage such behavior, we used a loss-framed incentive (ie, a deposit contract) that deducts estimated amounts during mission failures from budgets paid in advance. Combined with the loss-framed incentive, our incentive mechanism gradually increases the amount deducted if the user consecutively rejects the bids, whereas if the user is more likely to accept the bids and comply with behavioral missions, the amount deducted for mission failures decreases. In such a mechanism, the optimal strategy for obtaining as many incentives as possible is to maintain a healthy behavior (eg, regularly interrupting prolonged sedentariness) to keep behavioral missions (which are designed to deduct incentives from the budget) from being triggered and comply with behavioral missions regardless of incentive amounts if missions are triggered. Not only does this strategy keep budgets without deduction, but it also decreases the amount deducted for mission failures because of unavoidable reasons.

In addition to the prevention of gaming behavior, another reason for using the loss-framed incentive is that the loss-framed incentive is more likely to elicit behavior change than the gain-framed incentive because of people’s tendency to place a greater emphasis on losses than gains, as stated by the prospect theory [43]. In practice, previous studies have demonstrated a better effect of the loss-framed incentive on health outcomes than the gain-framed incentive in a variety of intervention domains, including mitigating smartphone overuse [23], promoting physical activity [35], and improving driving behavior [26].

### Implementation of Mobile Health Intervention

#### Overview

**Figure 2:**
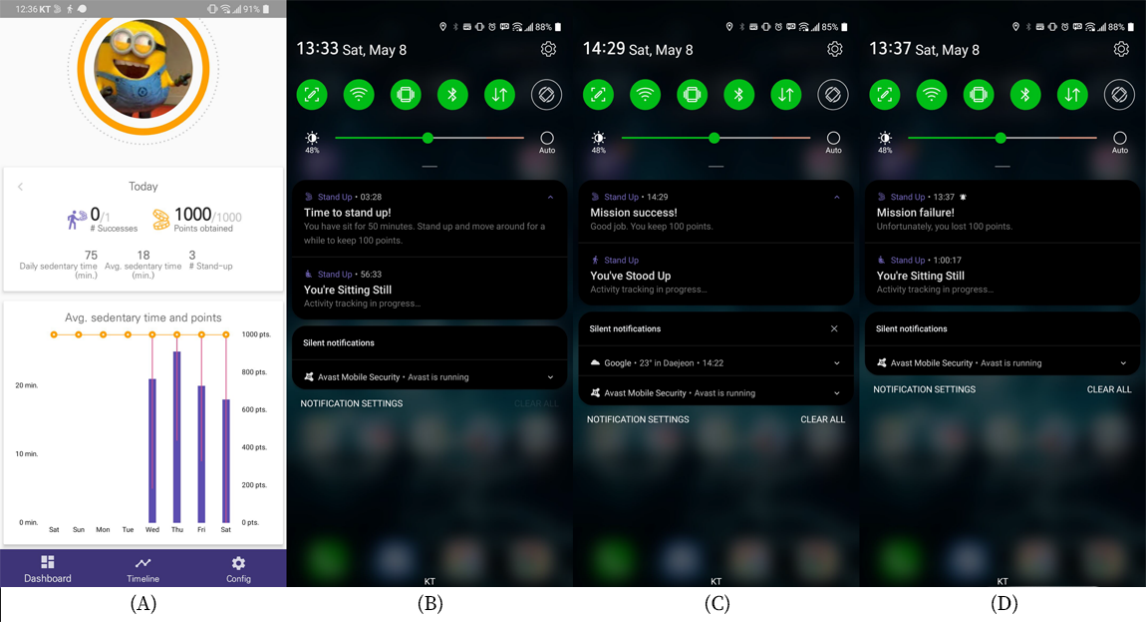
Overview of the StandUp user interface. From the left, (A) dashboard summarizing mission results and compensation, (B) mission trigger notification, (C) mission success notification, and (D) mission failure notification.

To explore how users respond to the proposed incentive strategy, we implemented a research app prototype named *StandUp*. This prototype comprises 4 major components: sedentary behavior tracking, context sensing, incentive estimation, and active break mission delivery. The StandUp user interface is presented in Figure 2.

#### Sedentary Behavior Tracking

Our prototype app monitors step counts through the user’s smartphone to detect sedentary behavior. The prototype assumes that the user is stationary when <10 steps are recorded in 1 minute. Moving or taking an active break is defined as >45 steps being recorded within a minute. In contrast, we considered that 10 to 45 steps within a minute were transitions between sedentariness and movement (or vice versa); hence, we refrained from exactly determining whether the user is sedentary in these cases. Specifically, 10 and 45 steps correspond to approximately 6.6 m to 7.9 m and 29.7 m to 35.6 m of movement, respectively [44,45]. The rationale behind the hard-coded threshold for step counts (ie, 10 and 45 steps) was derived from an internal pilot test wherein these step numbers corresponded to walking for 30 to 60 seconds, respectively. StandUp schedules an intervention prompt to appear after 50 minutes when users become stationary. In addition, the scheduled prompt is canceled if a substantial movement change (ie, at least 10 steps within a minute) is detected.

#### Context Sensing

Context sensing is used for tailoring incentives to different contexts. As location substantially contributes to users’ decisions to comply with interventions [40], our prototype considers location as the key context variable. Once any stationary event occurs, StandUp retrieves the latitude and longitude of the current location from the smartphone’s GPS sensor.

#### Active Break Mission Delivery

If there is no mobility state change for 50 minutes after an intervention prompt has been scheduled, a user receives a mission that suggests taking an active break in the form of a smartphone notification. Each mission lasts 10 minutes and informs about a specific expense deducted from a budget upon failing that mission, where the budget is individually assigned at the start of every day. The 10-minute threshold for adherence to the mission is based on the finding that people see incoming smartphone notifications within 10 minutes on average from notification arrival even when the ringer mode is set to silent [46].

After the notification appears on the smartphone, StandUp begins to check via sedentary behavior tracking whether a user takes an active break within 10 minutes. If a given mission expires without behavior change (ie, no mobility is detected within 10 minutes of mission delivery), StandUp reminds the user of the amount lost via a notification and deducts the amount from the user’s budget. Otherwise, a message of mission success is displayed on the notification. In the case of mission failures, StandUp reschedules the next active break mission to be delivered after 50 minutes. After each mission is completed, StandUp records the mission result (ie, success vs failure), amount of suggested expenses, and GPS coordinates of the current location. These data are stored in the user’s smartphone’s internal storage and used for incentive estimation in subsequent missions. In addition, they are later uploaded to a server via the Wi-Fi network.

#### Incentive Estimation

StandUp supports either a fixed or adaptive incentive strategy. StandUp with a fixed incentive strategy presents a predefined expense without any estimation. Regarding the adaptive incentive strategy, StandUp obtains the mission results (ie, success or failure), expense bids, and GPS coordinates of the locations where missions were initiated within the most recent 7 days. The continuous GPS coordinates should be transformed into discrete factors for modeling user behaviors in response to varying expenses and locations. We used a geohash for this purpose, which maps all locations on Earth onto rectangular grids and represents each rectangle as a short alphanumeric string. Our implementation maps GPS coordinates within 150-m by 150-m square grids to a single 7-character geohash string (ie, a 7-bit geohash) so that continuous GPS coordinates are discretized. Geohashed representations of locations are then factored with one-hot encoding. Consequently, mission results, expense bids, and factored locations were used for user behavior modeling and incentive estimation (Multimedia Appendix 1). In addition, the current implementation sets the probability of expected behavior occurrence (ie, y ¯) to 0.5. Such a parameter may allow the adaptive incentive strategy to actively explore the smaller incentive magnitude that is potentially optimal for eliciting behavior change.

### Study Design

For 3 weeks, we conducted a single-blind, between-group study with 2 groups: fixed incentive and adaptive incentive. All participants received US $1.50, which is presented as 100 points in StandUp, as a daily budget each morning during the intervention period. We used this specific value (US $1.50) as it is the median value of the daily incentives used in previous studies on incentive interventions for improving physical activity [31]. The fixed incentive group lost US $0.30 whenever participants failed a given active break mission. In the adaptive incentive group, participants lost an amount of incentive estimated by the proposed incentive strategy, in which the incentive ranged from US $0.30 to $3 with a US $0.30 increment (namely, US $0.30, US $0.60,..., US $2.70, and US $3) and the closest to the estimated one within that range was bid. For example, if the estimated incentive was US $1.40, the real incentive presented to users was US $1.50. If the daily budget was exhausted, participants did not receive any incentives on that day. The field trial was conducted between April 2020 and May 2020.

### Recruitment and Procedures

We recruited participants from our web-based campus community and Facebook. The inclusion criteria were as follows: having a sedentary occupation, spending >6 hours sitting on weekdays, and possessing a smartphone with an Android version 7.00 or higher. The participants were randomly assigned to the fixed and adaptive groups so that there was no significant difference between the groups regarding demographics such as age (*P*=.72; *t*_38.238_=0.363) and gender (*P*>.99; N=41, *χ*^2^_1_<0.0). Before participating in the field study, they received information on the health risks of prolonged sedentary behavior and how to use StandUp. In addition, we briefly instructed participants in the adaptive group on how incentive amounts were estimated (eg, as they become less likely to adhere to behavioral missions, a larger deducted amount is presented). However, we did not explain the detailed algorithm underlying our incentive mechanism (eg, mathematical equations describing user behaviors in response to incentives and contexts) as we believed it might be difficult for the general population to comprehend.

The first week was the baseline period, with StandUp just displaying the minutes that participants spent in a sedentary state on its dashboard and not delivering any active break missions. This period was intended to minimize the novelty effect of our app on any user behavior. After the baseline period, through SMS text messages, we asked participants to activate the mission delivery option for the second and third weeks. Participants were allowed to choose the start time of the missions from 9 AM to 11:59 AM depending on their preferences. The mission prompts were delivered over 9 hours from the chosen start time (eg, 9 AM-6 PM to 11:59 AM-8:59 PM) every day during the intervention period. Thus, at most, 10 missions were delivered to participants per day if they remained sedentary during the mission activation period.

After the field study, we compensated participants with US $24 for study participation and extra payments for the results of their missions (US $21 extra at maximum). In addition, exit interviews lasting 30 minutes were conducted with each participant to investigate user experiences with StandUp and potential factors relevant to the effectiveness of different incentive strategies.

### Exclusion Criteria

To clean the data, we first excluded missions collected at the first date of the intervention period as participants manually activated the active break mission delivery option on the first day of the intervention period (the eighth day of the entire field study) and the missions collected on that date possibly contained noise. In addition, we found that StandUp did not operate for a few days for several participants, resulting in a large loss of mission results. Therefore, we excluded all missions from participants whose data did not show any missions triggered for 2 consecutive days.

### Measurements and Data Analysis

The major outcome for evaluating the effectiveness of the proposed incentive strategy was the success rate of active break missions. It was defined as the ratio of the number of successful missions to the number of missions triggered. On the basis of the results of the Shapiro-Wilk normality test, both groups’ success rate was normally distributed (for the fixed incentive group: *P*=.08, and *W*=0.905; for the adaptive incentive group: *P*=.51 and *W*=0.953). Therefore, we compared the means of the success rates of the fixed and adaptive incentive groups using the Welch 2-tailed *t* test, which is known to have better control over type-1 errors than the Student *t* test [47].

In addition, we performed follow-up analyses of the adaptive incentive group to investigate in depth the effects of various factors on the mission success rate. First, we conducted a generalized linear mixed model (GLMM) analysis, hypothesizing that the mission success rate may be affected by days passed since the intervention onset, expense bids, and location. A reason for including the days passed since the intervention onset in the GLMM analysis is that repeated provision of intervention prompts during intervention periods would decrease responsiveness to those prompts because of the habituation effect [48]. Other 2 factors, deducted amounts and location, were examined to corroborate a hypothesis regarding our incentive mechanism, namely, that the occurrence of the target behavior would vary by context and incentive.

Before building the GLMM, we preprocessed the location data. First, we converted the GPS coordinates of locations where missions were triggered into 7-bit geohash strings, as our incentive mechanism did. As our participants resided elsewhere, geohashed locations would also be different and, thus, could not be used in the GLMM as a factor. Therefore, we relabeled geohashed locations considering the number of behavioral missions triggered (ie, the number of times prolonged sedentariness happened) at each location. For example, the top-*k* location refers to the geohashed location where missions were the *k*th most frequently triggered out of all geohashed locations. We analyzed the top 5 locations where 90% of the missions were triggered. Consequently, our GLMM included the following fixed effects—expense bids, days passed since the intervention onset, and the top 5 locations where missions were triggered—and the following random intercepts—the participants and top 5 locations within participants (ie, nested random effects). A detailed formula for the GLMM is presented in Multimedia Appendix 2.

Another follow-up analysis was conducted to examine the distribution of coefficients in the hypothetical user behavior model (ie, β_0_), which was updated for each behavioral suggestion, to investigate whether our incentive estimation worked as intended.

### Ethics Approval

This study was approved by the institutional review board of the Korea Advanced Institute of Science and Technology (KH2019-114), and we obtained written consent from all participants.

## Results

### Population Characteristics

A total of 41 participants initially took part in the 3-week field trial. The mean age was 24.0 (SD 3.8; range 19-34) years, and there were 37% (15/41) female participants. Most participants were graduate (17/41, 41%) and undergraduate (20/41, 49%) students. The other 10% (4/41) of participants were an office clerk, a graphic designer, a private academy instructor, and a researcher. In addition, of the 41 participants, 20 (49%) and 21 (51%) were assigned to the fixed and adaptive incentive groups, respectively. In total, 2387 missions (n=1021, 42.77% failures) were recorded during the field trial. From the data cleaning, of the 2387 missions, we excluded 179 (7.5%) collected on the first day of the intervention period and 399 (16.72%) from 7 participants for whom StandUp did not operate well. The following analyses were conducted with the remaining 1809 missions (n=684, 37.81% failures) from 34 participants (n=13, 38% female participants; n=17, 50% of participants in each group) whose mean age was 24.2 (SD 4.0; range 19-34) years.

### Comparison of Mission Success Rate

Participants in the fixed and adaptive incentive groups received 900 (n=347, 38.6% failures) and 909 (n=337, 37.1% failures) active break missions, respectively. Although the fixed incentive group (mean 0.66, SD 0.23) showed a larger success rate than the adaptive incentive group (mean 0.61, SD 0.22), the Welch *t* test showed that the difference between the groups was not statistically significant (*P*=.54; *t*_31.85_=0.62).

### Follow-up Analysis of the Adaptive Incentive Group

#### GLMM Analysis

As noted previously, most missions in the adaptive group were triggered at the top 5 locations (831/909, 91.4%), in which the top-1 to top-5 locations occupied 60.3% (548/909), 15.4% (140/909), 7.7% (70/909), 4.8% (44/909), and 3.2% (29/909) of the missions, respectively. As shown in Table 1, the GLMM analysis revealed that expense bids did not show statistical significance on mission success (*P*=.18; odds ratio [OR] 0.98, 95% CI 0.95-1.01). Meanwhile, the location where missions were fourth most frequently triggered (*P*=.04; OR 0.49, 95% CI 0.25-0.96) and the days passed since the intervention onset (*P*=.03; OR 0.95, 95% CI 0.91-0.99) had a statistically significant influence on mission success.

**Table 1:** Results of the generalized linear mixed model analysis for behavior occurrence likelihood in the adaptive incentive group. Marginal and conditional *R*^2^ are 0.030 and 0.312, respectively.

Fixed effects		β (SE)	*z* score	OR^a^ (95% CI)	*P* value
Intercept		1.32 (0.46)	2.90	3.75 (1.53-9.18)	.004
Expense bids		−0.02 (0.02)	−1.33	0.98 (0.95-1.01)	.18
**Top-*k* location**					
	Top 1	0.24 (0.22)	1.07	1.26 (0.82-1.96)	.29
	Top 2	−0.18 (0.25)	−0.73	0.83 (0.51-1.37)	.47
	Top 3	0.02 (0.30)	0.06	1.02 (0.57-1.82)	.96
	Top 4	−0.71 (0.34)	−2.08	0.49 (0.25-0.96)	.04
	Top 5	0.71 (0.42)	1.66	2.03 (0.88-4.65)	.10
Days since intervention onset		−0.05 (0.02)	−2.23	0.95 (0.91-0.99)	.03

^a^OR: odds ratio.

#### Coefficients Corresponding to Incentive Magnitudes

**Figure 3:**
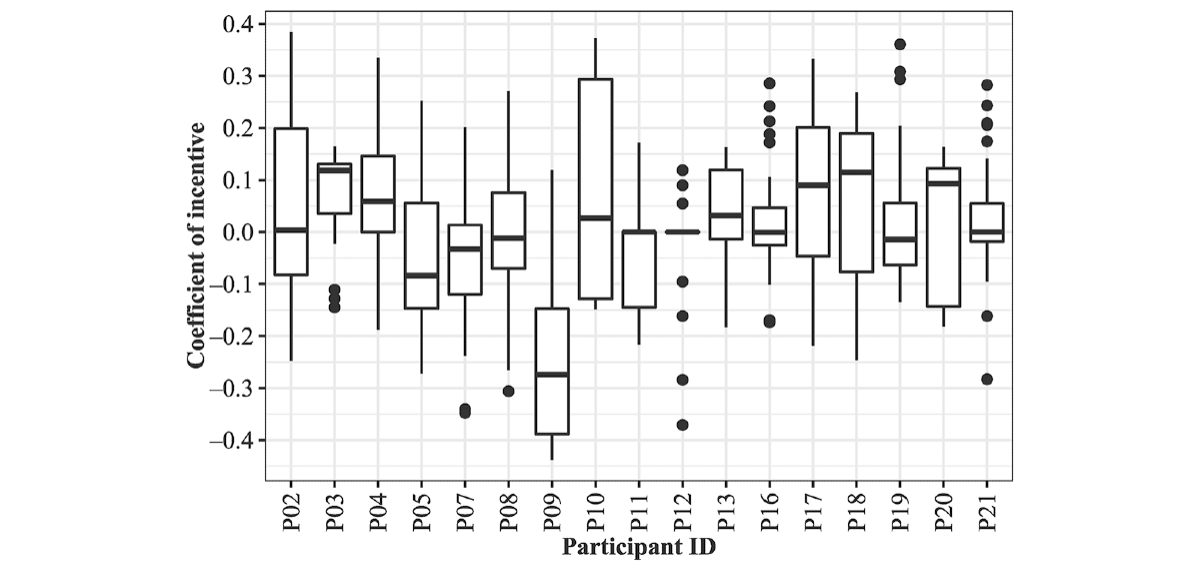
Distribution of coefficients corresponding to the incentive magnitude across participants.

We further investigated how the proposed incentive strategy estimated the effect of the incentive magnitude for every expense bidding. As shown in Figure 3, we found that β_0_ often became negative where the mean of the coefficient across participants was 0.00 (SD 0.08; 95% CI −0.04 to 0.04). In other words, our incentive strategy often estimated that bidding larger expenses rather inhibited participants’ behavior occurrence likelihood.

#### Corroborating Statistical Analyses via Interviews

Although our statistical analysis did not show a clear relationship between mission success rate and incentive magnitudes, we discovered 2 major behavioral patterns related to incentives in the qualitative interview analysis. One pattern was that participants randomly accepted behavioral suggestions regardless of the expenses offered. Some participants described being intrinsically motivated toward engaging in the active break as they were aware of the risk of sedentary lifestyles or had already felt that their sitting time was too long. These participants typically tried to accomplish active break missions without checking how much expense was bid, as participant 4 noted:

I’m having lower back pain when I sit down and keep myself focused on studying for an hour or two. While using this app (StandUp), I stood up every 50 minutes and felt that my back pain was greatly alleviated. The main reason I followed the active break suggestions was for health benefits, not the money.

Meanwhile, a participant (participant 8) reported choosing to adhere to the mission after 50 minutes of being in a sedentary state regularly to improve his productivity, in a method similar to the Pomodoro technique:

Perhaps the original purpose of this app (StandUp) is to prevent some cardiovascular diseases by increasing physical activity. However, I used this app for a different reason; I used to be less efficient when focusing on one thing. Once I engaged in an active break mission, I organized my thoughts for a while as I walked. So, I felt that my productivity improved.

Another pattern was that participants adhered to behavioral suggestions only when substantial expenses were presented. Subsequently, participants tended to be less sensitive to minor changes in expenses. Several participants had different criteria for the minimum expense that made them consider mission acceptance. Therefore, these participants tended to reject missions when incentives smaller than their criteria were offered, as participant 5 noted:

I tried to accept missions when this app (StandUp) will take back at least 0.15 USD. Such an amount is like my psychological Maginot line.

## Discussion

### Principal Findings

Financial incentives have been widely regarded as effective behavior reinforcers in diverse health and behavior change domains [31,33,49-54]. However, to the best of our knowledge, most previous studies on incentive-based health interventions often assumed that users’ responses to incentives were homogeneous; thus, compensation for positive behavior changes was fixed even in different individuals and contexts. In addition, these studies required intervention providers to manually configure incentive strategies based on their domain knowledge. Meanwhile, this study argues against such one-size-fits-all incentive strategies and explores the feasibility of a novel incentive strategy, L-AMCM. It personally and contextually tailors the incentive magnitude to users, which is then immediately suggested to them to reinforce behavior changes. We hypothesized that we could computationally learn about an individual’s preference for incentives by referring to one’s previous responses to incentives in varying contexts.

We developed a simple mobile health intervention targeted at discouraging prolonged sedentary behaviors by delivering information for users about prolonged sedentariness (ie, 50-minute sitting sessions) and by suggesting, via app notifications, that they take active breaks with a loss-framed, low-cost financial incentive. We assumed that people would be more likely to adhere to the behavior suggestions as the deducted amount grew; this led to an LR-based context-aware incentive adaptation where the deducted amount dynamically changed depending on adherence to the behavioral suggestion across different incentives and contexts. Unfortunately, our 3-week between-group field trial with 41 participants showed that the proposed incentive strategy failed to promote more adherence to health behaviors than a fixed incentive strategy. Furthermore, the follow-up analyses partially confirmed that location influenced behavior occurrence likelihood. However, it was found that behavior occurrence likelihood did not always increase as incentive magnitude increased, possibly because of enough intrinsic motivation and less sensitivity to incentive magnitude.

### Lessons Learned

From our findings, we learned lessons that may improve incentive magnitude tailoring for contingency management interventions. As a previous study pointed out that the effect of incentive magnitude on decision-making is small [55], one lesson is that acceptance of behavioral suggestions may not be proportional to incentive magnitude; namely, our results were not concordant with our initial expectations. At least in the sedentary behavior intervention we designed, there may be a case in which users may accept or reject behavior suggestions without regard to incentive magnitude. Although somewhat counterintuitive and radical, we may design different incentive strategies where a user’s behavior occurrence likelihood and incentive magnitude are independent of each other instead of there being a linear relationship between them.

Another lesson is that incentive magnitudes should change in a coarse-grained manner. In this study, incentive magnitudes were set to range from US $0.30 to $3 with a US $0.30 increment; nonetheless, participants reported in the interviews that they were less sensitive to fine-grained changes in incentive magnitudes and that they instead had a rough threshold for considering adherence to behavioral suggestions. Hence, substantial changes in incentive magnitude may be more appropriate for eliciting differential behavioral patterns.

### Limitations and Future Work

Our sedentary behavior tracking used step counts obtained from an individual’s smartphone; thus, it has constraints such as requiring participants to always carry their smartphones and move around at least 30 m to detect active break sessions. Unfortunately, these constraints made it impossible to capture behavior change when participants did not carry their smartphones and to differentiate a standing activity that can interrupt sedentariness (eg, standing and stretching or working at a standing desk) from sedentary behavior. Detecting sedentary activity by identifying an individual’s posture (eg, lying, sitting, or upright position) with wearable sensors (eg, a thigh-attached accelerometer) could be an alternative for tracking sedentary behavior with better precision and granularity [56].

Another limitation of this study was that health-related outcomes were not measured. Given that previous studies have demonstrated the health benefits of contingency management [52] and prompt-based interventions [57], we assumed that our prompt-based contingency management intervention probably had a positive impact on health outcomes in our experimental design phases. Under such an assumption, the primary objective of this study was to compare different incentive mechanisms in terms of the occurrence of the desired behavior and not to confirm the general health effects of the proposed intervention. Nonetheless, it would be beneficial to precisely measure health-related outcomes such as time spent in sedentary or physical activity [57,58] to establish not only the general health benefits of our intervention but also to rigorously compare the effects of various incentive mechanisms.

For ease of implementation, the incentive mechanism presented in this study used only geohashed locations as contextual factors. Unfortunately, it is challenging to interpret geohashed locations intuitively; thus, our GLMM analysis only partially confirmed the impact of location on behavior occurrence and did not provide a comprehensive interpretation of these locations. It would be beneficial to assign semantic meaning to geohashed locations (eg, home, workplace, or eatery) to clearly understand which attributes of locations influence behavior occurrence. For example, a future study may ask participants to name their locations semantically after delivering intervention prompts via ecological momentary assessment [40,56]. In addition, there would be other contextual factors (eg, ongoing tasks and social settings [40]) and intrinsic attributes (eg, self-efficacy and perceived enjoyment [59] and affective responses [60] toward the target behavior) that may influence an individual’s response to incentives. Future work may try modeling user behavior with several variables as their results will probably improve our knowledge of appropriate incentive magnitude estimation for contingency management interventions.

Although this study does not reveal the benefits of the L-AMCM in terms of target behavior occurrences over a short intervention period, a long-term and follow-up investigation might disclose intriguing effects on user behaviors, supposing that the L-AMCM works as intended. The mitigation of habituation is one of the potential effects we expect. As with the fixed incentive mechanism, the repeated provision of monotonous incentives (ie, providing stimuli with the same intensity) may diminish the perceived value of incentives over time [61]. In contrast, the unique nature of the L-AMCM to offer varying incentives based on users’ responsiveness to incentives (ie, providing stimuli with varying intensities) may keep users from becoming accustomed to intervention prompts to some extent.

Furthermore, it would be an interesting research direction to design multicomponent interventions, including incentive adaptation, by considering challenges specific to sedentary behavior. Previous studies have found, for example, that people tend to identify sedentary behavior as a behavior entailing sitting rather than sedentary behavior itself [62]. Hence, sedentary behavior may be habitual and not purposeful [63], and the time spent in sedentariness was found to be underestimated [64]. This lower awareness of sedentary behavior may make people less aware of its adverse health effects and the health benefits of breaking up long periods of sedentary behavior, possibly leading to decreased motivation toward interventions for preventing sedentary behavior. As an example of how to make people aware of the health risks associated with prolonged sedentariness, the intervention might provide information on behavioral consequences [65]. For example, our mission prompt can be designed to convey specific health outcomes that may result from accepting or rejecting behavioral missions (eg, “Taking an active break now can reduce the risk of a cardiovascular disease by XX%”).

This feasibility study considered prolonged sedentary behavior, which can be easily detected with an off-the-shelf smartphone [40,57], to avoid technical challenges irrelevant to the L-AMCM. However, we believe that the L-AMCM might be used in a wide range of intervention domains that satisfy certain criteria. The first (but not mandatory) criterion is that unhealthy behavior needs to happen somewhat frequently so that responses to incentives are collected to some extent within a short period and a behavior model is quickly learned. The other criterion is that health behavior or outcome changes should be monitored following the bidding of incentives to track responses to particular incentives. For example, smoking cessation would be a suitable intervention domain for applying the L-AMCM; smoking episodes occur frequently and can be automatically detected despite some technical challenges (eg, requiring multiple sensor units such as a wrist-worn sensor for detecting wrist-to-mouth movements and a chest-worn sensor for examining inhalation and exhalation [66]).

### Conclusions

This study aimed to devise a novel incentive strategy that adjusts incentive magnitude depending on individuals’ behaviors in different contexts and incentives, as well as explore the feasibility of such a strategy via a field trial. To this end, we first developed the LR-based incentive estimation with the expectation that behavior occurrence likelihood would vary by incentive magnitude and context and increase as the incentive grows. However, the 3-week field study showed that users’ actual behaviors were nonconcordant with our expectations. Thus, the proposed incentive strategy showed no statistically significant differences from the fixed incentive strategy. Interestingly, the follow-up analyses revealed that users might be less sensitive to minor changes in incentive magnitudes. Although the proposed incentive strategy failed to show its effectiveness clearly, we believe that this study was the first step toward incentive adaptation for mobile health interventions and hope that it inspires various other researchers to develop and test adaptive incentive strategies.

## Appendix

Multimedia Appendix 1 An algorithm of a logistic regression–based incentive estimation.

Multimedia Appendix 2 A generalized linear mixed model formula.

## Abbreviations

GLMM: generalized linear mixed model
L-AMCM: loss-framed adaptive microcontingency management
LR: logistic regression
OR: odds ratio
