# Supplementary figures and images for: Loss-Framed Adaptive Microcontingency Management for Preventing Prolonged Sedentariness: Development and Feasibility Study
Source: JMIR Mhealth Uhealth. 2023 Jan 27;11:e41660. doi: 10.2196/41660 (PMC9919499; doi:10.2196/41660)

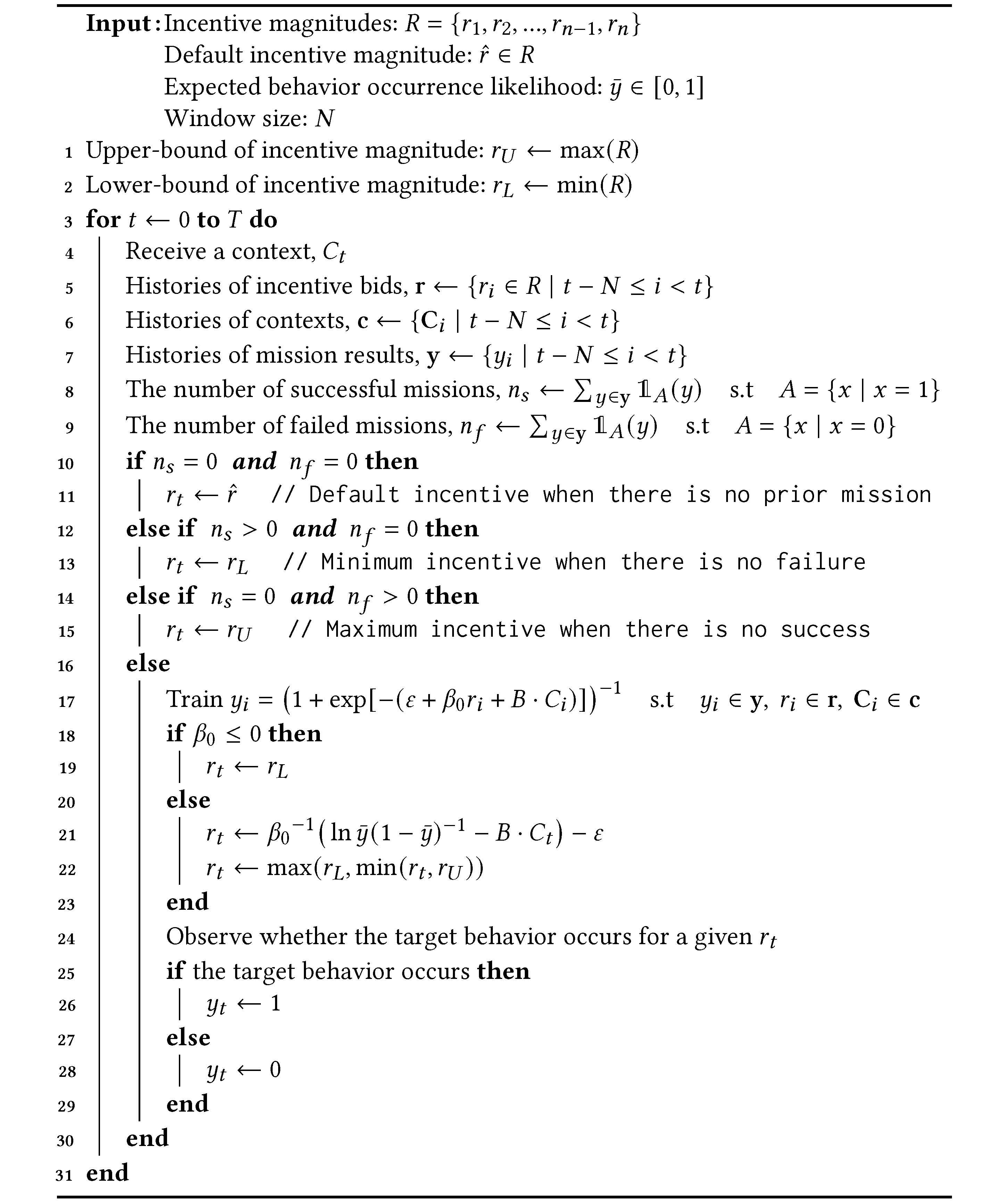

Supplement: Multimedia Appendix 1 [file mhealth_v11i1e41660_app1.png]

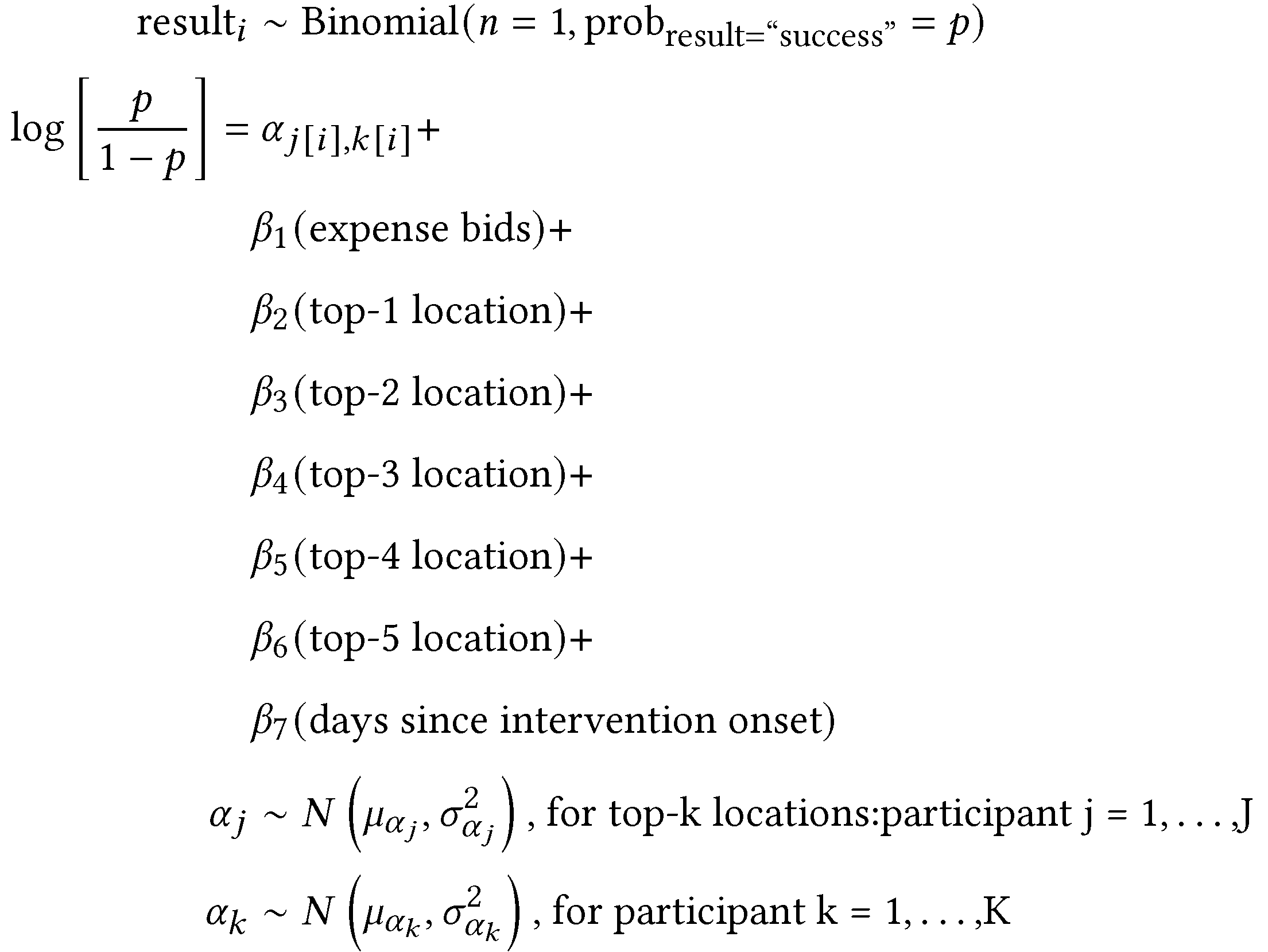

Supplement: Multimedia Appendix 2 [file mhealth_v11i1e41660_app2.png]
